# Elucidating synergistic dependencies in lung adenocarcinoma by proteome-wide signaling-network analysis

**DOI:** 10.1371/journal.pone.0208646

**Published:** 2019-01-07

**Authors:** Mukesh Bansal, Jing He, Michael Peyton, Manjunath Kustagi, Archana Iyer, Michael Comb, Michael White, John D. Minna, Andrea Califano

**Affiliations:** 1 Psychogenics Inc., Paramus, New Jersey, United States of America; 2 Department of Neuroscience, Icahn School of Medicine at Mount Sinai, New York, NY, United States of America; 3 Department of Systems Biology, Columbia University, New York, NY, United States of America; 4 Center for Computational Biology and Bioinformatics (C2B2), Columbia University, New York, NY, United States of America; 5 Department of Biomedical Informatics (DBMI), Columbia University, New York, NY, United States of America; 6 Department of Cell Biology, University of Texas Southwestern Medical Center, Dallas, TX, United States of America; 7 Cell Signaling Technology, 3 Trask Lane, Danvers, MA, United States of America; 8 Hamon Center for Therapeutic Oncology Research, Simmons Comprehensive Cancer Center, Departments of Pharmacology, and Internal Medicine, University of Texas Southwestern Medical Center, Dallas, Texas, United States of America; 9 Department of Biochemistry and Molecular Biophysics, Columbia University, New York, NY, United States of America; 10 Institute for Cancer Genetics, Columbia University, New York, NY, United States of America; 11 Herbert Irving Comprehensive Cancer Center, Columbia University, New York, NY, United States of America; H. Lee Moffitt Cancer Center & Research Institute, UNITED STATES

## Abstract

To understand drug combination effect, it is necessary to decipher the interactions between drug targets—many of which are signaling molecules. Previously, such signaling pathway models are largely based on the compilation of literature data from heterogeneous cellular contexts. Indeed, *de novo* reconstruction of signaling interactions from large-scale molecular profiling is still lagging, compared to similar efforts in transcriptional and protein-protein interaction networks. To address this challenge, we introduce a novel algorithm for the systematic inference of protein kinase pathways, and applied it to published mass spectrometry-based phosphotyrosine profile data from 250 lung adenocarcinoma (LUAD) samples. The resulting network includes 43 TKs and 415 inferred, LUAD-specific substrates, which were validated at >60% accuracy by SILAC assays, including “novel’ substrates of the EGFR and c-MET TKs, which play a critical oncogenic role in lung cancer. This systematic, data-driven model supported drug response prediction on an individual sample basis, including accurate prediction and validation of synergistic EGFR and c-MET inhibitor activity in cells lacking mutations in either gene, thus contributing to current precision oncology efforts.

## Introduction

Lung adenocarcinoma (LUAD) is a leading cause of cancer related deaths in United States, representing 40% of 225,500 new lung cancer cases every year, and has a 5-year survival rate of only 16% [1]. Excluding immunotherapeutic agents, which have recently shown significant success in a relatively small subset of patients [2], the most effective targeted therapies for this diseases were designed to inhibit tyrosine kinase proteins harboring genetic alterations that induce aberrant activation of downstream pathways [3–7]. Among these, the most frequent actionable alterations include EGFR mutations and EML4-ALK fusion events, in ~15% and ~3–7% of LUAD patients, respectively [8, 9]. Yet, while targeted therapy is initially effective in a significant fraction of tumors harboring these genetic alterations, the vast majority of treated patients will either fail to respond or will develop resistance to mono-therapy [10, 11]. In addition, most patients lack actionable alterations altogether. This suggests that novel approaches are critically needed.

A possible alternative to minimize emergence of resistance is combination therapy, a strategy that has been shown to be effective in many metastatic tumors, such as breast cancer and acute myeloid leukemia [12–14]. However, systematic identification of effective drug combinations on a genetic alteration basis is difficult, because the number of patients presenting multiple actionable events is extremely low. As a result, combination therapy is generally hypothesized and tested on an empirical basis or based on elucidation of complex mechanisms of tumor cell adaptation. In addition, accurate prediction of response to available mono-therapy–including to EGFR inhibitors–in patients lacking any genetic alteration represents an equally relevant challenge, especially since a small fraction of EGFR^WT^ patients have been shown to respond to Afatinib [15], even though a predictive biomarker is not available. To address these limitations, we and other have proposed that rational design of combination therapy and the identification of critical targetable dependencies may require a more mechanistic and tumor-context-specific understanding of the molecular interactions that underlie their potential synergistic activities, starting with tyrosine kinases, which represent a critical class of pharmacological targets in cancer [16]. Such approach requires methodologies for the accurate and systematic elucidation of tumor-specific signaling transduction pathways.

Dissection of signal transduction networks represents a complex endeavor, requiring elucidation of thousands of tissue-specific molecular interactions that mediate the post-translational modification of protein substrates. *In vitro* approaches generally fail to capture the tissue-specific nature of these interactions, thus providing “average” signal transduction pathways that are both incomplete and inaccurate. In addition, experimental approaches that have been successful in accelerating the analysis of molecular interactions in transcriptional regulation and protein-protein interaction in stable-complexes, such as those based on co-expression or yeast-2-hybrid assays, do not easily translate to elucidate signaling interactions. Similarly, approaches based on the use of phospho-specific antibodies, while elegant and effective, are limited to only a handful of proteins. Computationally, compared with the many algorithms that have been developed for the reverse engineering of transcriptional and protein-complex interactions [17, 18], only a handful of experimentally validated algorithms are available for the dissection of signaling networks, none of which works at the proteome-wide level or is tumor-context specific [17, 19, 20].

Recent availability of proteome-wide molecular profile data, characterizing the abundance of phospho-tyrosine-enriched peptides by liquid chromatography coupled to tandem mass spectrometry (LC-MS/MS), suggests that additional methodologies may be developed to extend approaches that have been successfully applied to the dissection of transcriptional networks from gene expression profiling. In this manuscript, we propose innovating the Algorithm for the Reconstruction of Accurate Cellular Networks (ARACNe) [21] for the reverse engineering of signal transduction networks from large-scale phosphoproteomic profiles.

We applied pARACNe to the analysis of previously published, global tyrosine phosphoproteomic data from 245 lung adenocarcinoma (LUAD) samples, including 151 tumor samples, 46 cell lines, as well as 48 normal lung tissues [22]. The resulting network comprised 46 tyrosine kinases (TK) densely connected with 415 candidate substrates (including 377 proteins lacking any TK activity), representing the first global tyrosine phosphoproteomics, tumor-context-specific model for a TK signal transduction network, capturing both protein-specific and phospho-site specific events. We validated substrate predictions for two “hubs”, whose activity may play a key role in determining sensitivity to Erlotinib and Crizotinib, two FDA-approved drugs for LUAD, including the EGFR and c-MET tyrosine kinases by independent SILAC assays and database analysis, with >60% accuracy. Of particular note, the inferred TK-substrate network provided unique information about tyrosine kinase auto-phosphorylation events, either direct (cis) or via a second kinase (trans).

Analysis of the resulting TK-network–by extending the VIPER (Virtual Proteomics by Enriched Regulon analysis) algorithm [23], an established method for the inference of Master Regulator proteins–recapitulated established genetic determinants of LUAD and was effective in predicting sensitivity to Erlotinib and Crizotinib combination therapy. Predicted sensitivities were validated in an independent set of LUAD cell lines, the majority of which harbored no genetic alterations in the corresponding genes. Furthermore, predictions based on the analysis of the corresponding patient cohort were strongly supported by genomic information, suggesting potential value in using these analyses for the identification of effective combination therapies in precision oncology.

## Results

**Fig 1:**
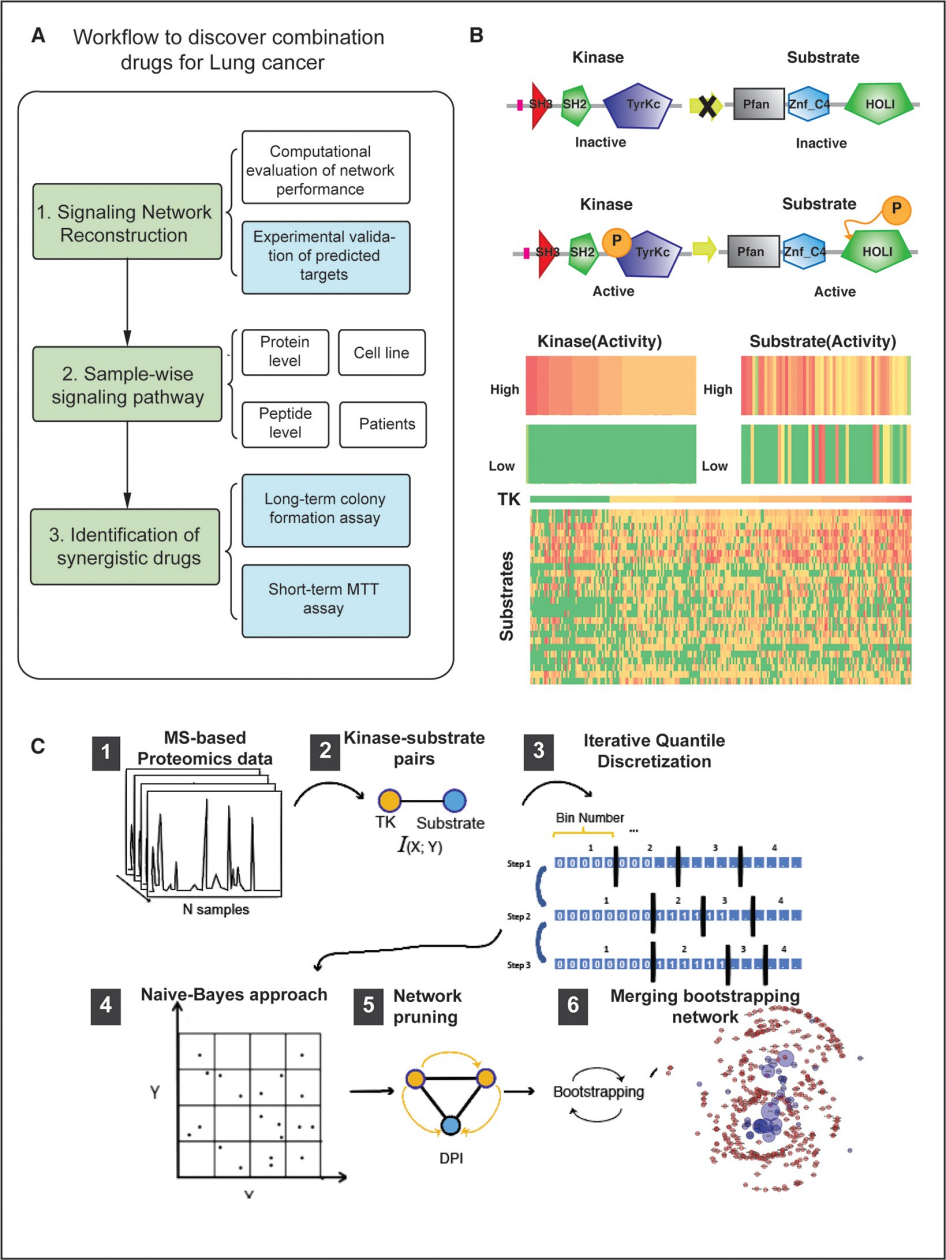
Framework for the reverse engineering of TK signaling networks from phosphoproteomic profiles. **(A)** Workflow to identify pharmacological synergistic dependencies based on context-specific signaling network analysis. **(B)** Top panel shows the schematic diagram of a **TK**→**S** interaction. The non-phosphorylated kinase is inactive in terms of phosphorylating a substrate, while the active isoform successfully phosphorylates the substrate. Bottom panel shows schematic diagram showing the correlation between TK phosphorylation and that of its potential substrates. The first two rows in the heatmap show proteins representing candidate TK substrates **(C)** Illustration of the pARACNe framework including 6 steps. Step-1 depicts peptides collection from primary lung cancer tissue and cell lines for whole phosphrtyrosine proteomics quantification. Step-2 depicts inferences of TK→S interactions using Mutual Information by Step-3 of the iterative quantile discretization methods and Step-4 Naïve-Bayes estimator. Step-5 and 6 depict network pruning and bootstrapping to construct final network. **(C)** Framework of pARACNe from LC-MS/MS data normalization, **I**terative **Q**uantile **D**iscretization (IQD) process, **M**utual **I**nformation (MI) calculation, **D**ata **P**rocessing **I**nequality (DPI) process, bootstrapping to network consolidation.

We started with construction of signaling network based on published global phosphoproteomic data (**Fig 1A, step 1**). To do this, we introduce a novel algorithm for the systematic inference of protein kinase pathways based on the phosphotyrosine proteomics profile data from 250 lung adenocarcinoma (LUAD) samples. Then, based on this signaling network, we used phospho-network based VIPER to identify candidate synergistic kinases for LUAD (**Fig 1A, step 2**). Finally, we validated the synergistic drug combinations identified in step 2 using both long-term and short-term drug treatment experiment (**Fig 1A, step 3**).

### Overview of the pARACNe algorithm

Enzymatic activity of tyrosine kinase (TK) proteins–as assessed by the ability to phosphorylate their downstream substrates–is effectively determined by their phosphorylated isoform abundance (**Fig 1B**). Therefore, we reasoned that computational inference of TK-substrate interactions (**TK**→**S**) could be effectively performed by measuring dependencies between their respective phospho-states by mutual information analysis [24] over a large sample compendium **(Fig 1C, step 1)**. Unfortunately, due to signal transduction cascade complexity and pathway cross-talk, such dependencies can manifest between protein pairs that are not involved in direct **TK**→**S** interactions. The ARACNe algorithm–previously designed for the reverse engineering of transcriptional networks–effectively addresses this problem by leveraging the Data Processing Inequality [25]. This is a critical property of the mutual information that effectively allows disambiguating between direct and indirect interactions by assessing whether information transfer on any candidate direct interaction (e.g., **TK**_1_→**S**) is greater than transfer on every other indirect path (e.g., **TK**_1_→**TK**_2_→**S**). ARACNe has been highly successful in the experimentally validated dissection of transcriptional networks via analysis of large gene expression profile compendia. ARACNe-inferred targets of transcription factors were validated in multiple cellular contexts, with an accuracy of 70% to 80% [21, 26–29].

However, ARACNe relies on molecular profile data that is both continuous and non-sparse, properties that are not always provided by quantitative proteomic data sets, which can be generated by a variety of methods. Those based on LC-MS/MS represent the most popular approaches [30], but different implementations have specific performance profiles in terms of analyte throughput, consistency of measurement of peptides across samples and linear dynamic range [31]. Depending on the data acquisition method, one or both of these assumptions of ARACNe are violated in proteome-wide datasets generated by the most popular methods based on data-dependent acquisition. Particularly when employing quantification by spectral counting, as is typically conducted for global protein-protein interaction studies [32, 33], phosphoproteomic data is both discrete (i.e., generally represented by spectral counts) and very sparse, with a majority of peptides having zero spectral counts and presenting a significantly skewed distribution for low-abundance peptides.

To address these limitations, we developed a phospho-proteomic specific algorithm, pARACNe (phospho-ARACNe) (**Fig 1C**), specifically designed to measure phospho-state dependencies between TKs and their candidate substrates from large-scale LC-MS/MS phosphoproteomic profiles. pARACNe thus extends the original ARACNe framework to allow systematic inference of **TK**→**S** interactions. Specifically, to handle the highly discrete nature of the data, we replaced the kernel-density and adaptive partitioning based mutual information estimators in the original algorithm with a bin-count based method (**Fig 1C, step 4**), using gold standard data to select the most effective number of bins [12] (see Methods). Furthermore, to deal with the skewed spectral count distribution, we introduce an iterative quantile discretization method, where samples are binned together, based on their spectral counts, to produce a distribution as close to uniform as possible (**Fig 1C, step 3,** Methods).

### pARACNe-inferred LUAD-specific TK-phosphorylation network

**Fig 2:**
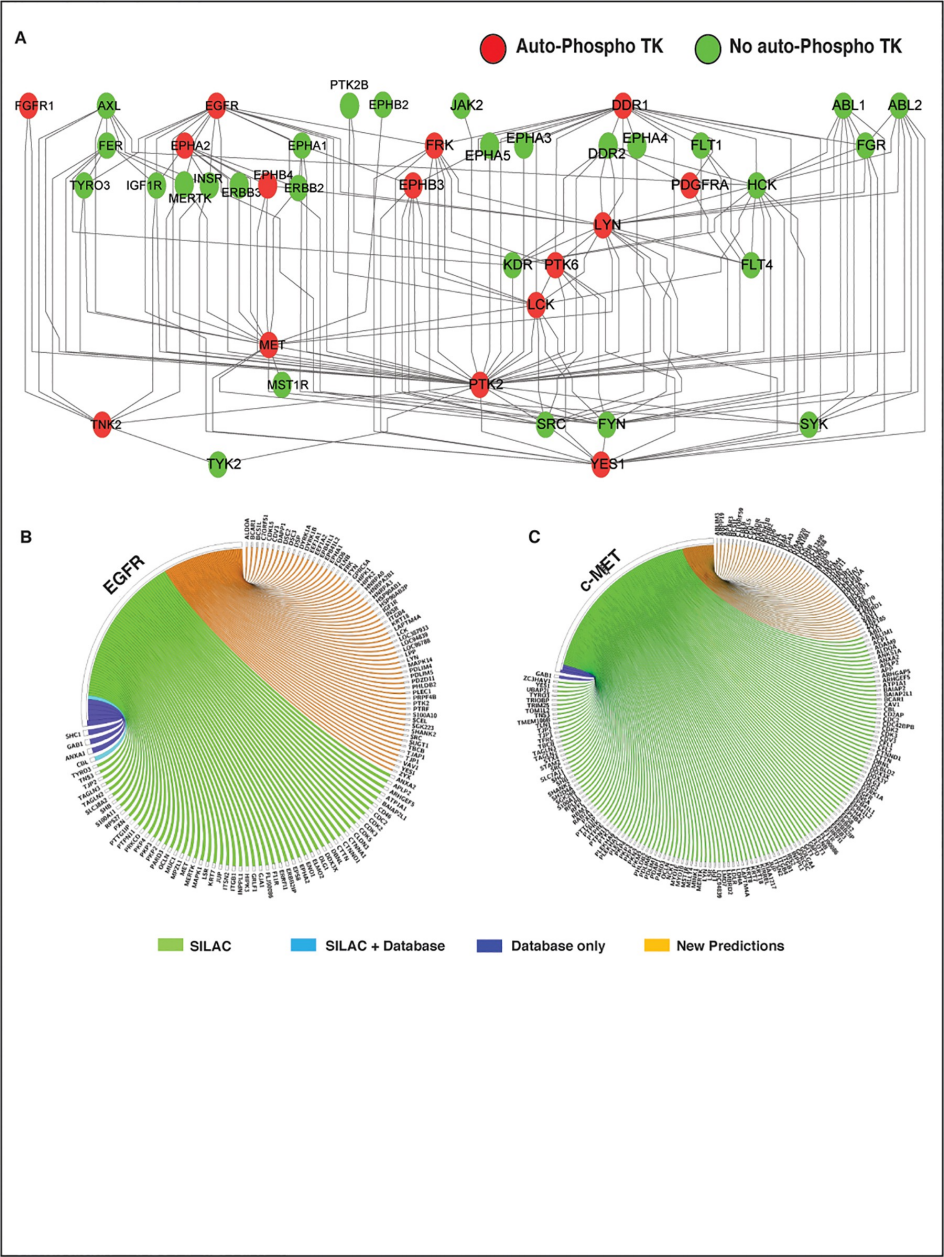
Predicted TK-TK network and validation of EGFR and c-MET prediction. **(A)** pARACNe-inferred densely inter-connected TK-TK network, with red nodes representing candidate TKs involved in auto-phosphorylation, where the phospho-state of a tyrosine is correlated with the phospho-state of a different tyrosine on the same TK protein. **(B)** pARACNe-inferred EGFR and **(C)** c-MET substrate overlap with SILAC-based and Database reported substrates, respectively.

We used pARACNe to reconstruct a LUAD-specific TK-signaling network, by analyzing phosphopeptide profiles obtained from 245 LUAD samples from Guo et al. [34]. These data represent the abundance of peptides containing at least one phospho-tyrosine, as obtained by phosphoproteomic analysis of 46 LUAD cell lines, 151 LUAD tumors, and 48 adjacent normal samples. LC-MS/MS profiling produced spectral counts for 3,920 phospho-tyrosine containing peptides mapping to ~2,600 different proteins. Based on these data, pARACNE identified 2,611 candidate phospho-peptide/phospho-peptide dependencies, which could be further mapped to 2,064 unique **TK**→**S** interactions (**S1 and S2 Tables)**. These represent interactions between 46 unique TKs and their candidate substrates. These include 174 **TK**_1_→**TK**_2_ interactions between two TKs (**Fig 2A**), representing a statistically significant bias toward TK-TK interactions in the network (*p* = 10^−62^). This suggests that, within the complete TK signaling network, TKs themselves may form a more densely inter-connected subnetwork than previously assessed, providing potentially valuable novel information about adaptive response, pathway cross-talk, and auto-regulatory loops.

Such highly interconnected structure provides potential functional advantage compared to less interconnected (i.e., “flat”) architectures, including the ability to provide more fine-grain response to a highly heterogeneous variety of exogenous signals and conditions, the ability to provide rapid adaptive response to changing stimuli, and the ability to preserve cell state via autoregulatory feedback. Consistent with the underlying biology, and in contrast to transcriptional networks, the vast majority of pARACNe-inferred interactions have a positive Spearman correlation, with higher counts of TK-mapped phosphopeptides corresponding to higher counts of candidate substrate-mapped ones. This is consistent with the fact that TKs only auto-phosphorylate or phosphorylate their substrates, thus inducing positive phospho-state correlation. Only a negligible number of inferred interactions (0.5%) were associated with a negative Spearman correlation (*N* = 11, *p ≤* 0.05). These may represent either indirect interactions where the TK activates a substrate-specific phosphatase or direct interactions where phosphorylation of one phosphosite may prevent phosphorylation of another site on the same protein.

### LUAD network accuracy and sensitivity analysis

To estimate the accuracy of the inferred TK-signaling network, we investigated the substrates of two TK-proteins, EGFR and c-MET, representing high-affinity binding targets of existing FDA-approved TK inhibitors for LUAD. Specifically, we compared their pARACNe-inferred substrates to those reported in the phosphoDB database [35] and those supported by experimental evidence, based on previously published SILAC assays, following cell line treatment with associated, selective TK inhibitors. pARACNe inferred 123 EGFR substrates (**Fig 2B**). Of these, 5 (blue and cyan) were included as high-confidence EGFR substrates in phosphoDB, out of 13 in total (38%), including the established EGFR auto-phosphorylation site. Moreover, 50 additional proteins (45%, green) showed significant decrease (at least 2 fold) in the abundance of their phosphorylated isoforms in SILAC assays [34], following treatment of H3255 cells with the EGFR inhibitor Gefitinib. Similarly, pARACNe predicted 179 c-MET substrates (**Fig 2C)**. Notably, both of the established substrates reported in PhosphoDB were identified by pARACNE (100%, blue). Moreover, 126 additional proteins (71.5%, green) showed significant decrease in the abundance of their phosphorylated isoforms in SILAC assays [34], following treatment of MKN45 cells with the first-generation c-MET-specific inhibitor Su11274. We used MKN45 to assess overall prediction accuracy **(Fig 2C)**, even though it represents a gastric cancer cell line, because signaling pathways such as EGFR-MAPK pathways proteins signaling are highly conserved across tissue and species [36]. Also, kinase interaction network structure and topology is conserved across spices [37]. Indeed, while lineage-specific chromatin state represents a major determinant of transcriptional regulation, it only affects signal transduction in terms of overall protein availability.

Taken together, these data suggest that pARACNe can identify a much larger subset of candidate substrates, while both identifying a significant proportion of established substrates (46% on average, based on phosphoDB) and maintaining high accuracy (~60% on average, by SILAC assays). This also suggest that, similar to transcription factor targets reported in the literature, TK substrates are still poorly characterized in existing repositories, even for highly relevant and exceedingly well-studied kinases such as EGFR and c-MET. As a result, pARACNe could provide significant novel hypotheses for TK→S interactions that can be validated as required. We should also note that the reported accuracy for pARACNe is estimated using SILAC data on a single cell line. SILAC assays, like all the other phosphorproteomics assays, have significant false negatives and it would be reasonable to expect that, once tested in additional cell lines, the accuracy of pARACNe could further increase. As a further performance benchmark, we used the same SILAC benchmark to test predictions by NetworkIN, a reverse engineering method based on protein sequence motif analysis and protein association networks [17]. The analysis found almost no consensus with SILAC assays, with only one out of 33 NetworkIN-predicted EGFR substrate identified as significantly dephosphorylated following treatment with TK-specific inhibitors.

### Systematic, network-based inference of pharmacological dependencies

**Fig 3:**
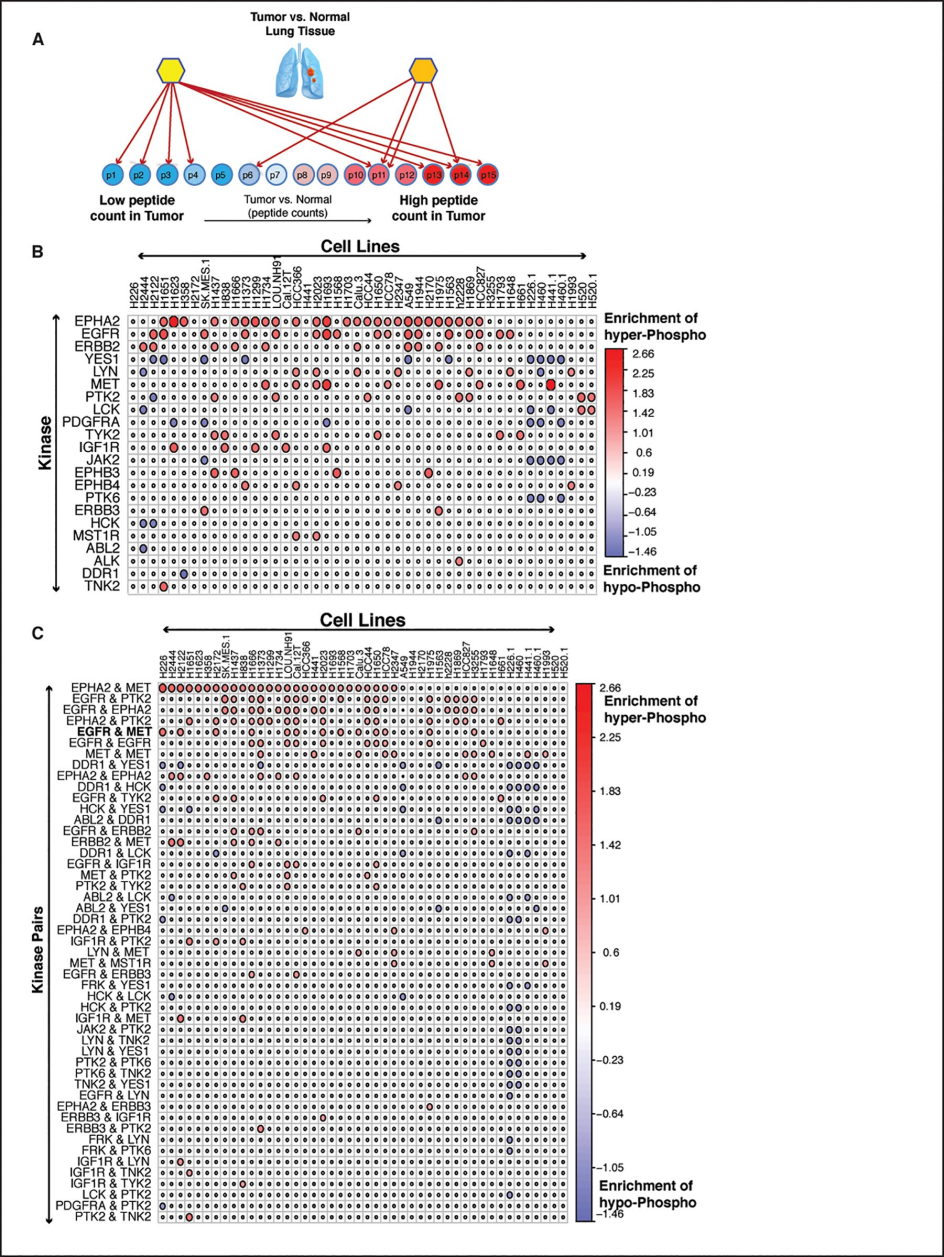
Inference of master regulator and combination. **(A)**. Schema of Master Regulator analysis in lung cancer using pVIPER. **(B)** shows prioritized Master Regulators and (MR) **(C)** shows prioritized Master Regulator Pairs as significantly activated (red circle) or de-activated (blue) molecules in different lung cancer cell lines (by column). In panel (B) and (C), pVIPER infers, for each MR / MR pairs in each cell line, the enrichment of the MR’s target peptides’ in the differentially phosphorylated peptides compared to the average of all normal samples. The size and color represent the scale of enrichment. Red color represents an enrichment of substrates hyper-phosphorylation by a Master Regulator or Master Regulator Pairs. Blue color represents that of hypo-phosphorylation. A white small circle refers to no significant enrichment; red (blue) large circle represents substrates of a kinase to be significantly hyper (hypo)-phosphorylated in the corresponding cell line when compared with the average from all normal samples.

Once an accurate model of signal transduction in LUAD cells was established by pARACNe analysis, we interrogated the corresponding TK→S network using phosphoproteomic signatures from 46 LUAD cell lines to identify key dependencies for experimental validation. For this purpose, we extended the VIPER algorithm (Virtual Proteomics by Enriched Regulon analysis)[23], which was originally developed to identify the Master Regulator (MR, a protein that regulate a collection of network substrates [28]) proteins that mechanistically regulate the transcriptional state of a tumor by assessing the enrichment of their transcriptional targets in differentially expressed genes in the tumor signature. VIPER and its predecessor MARINa (Master Regulator Inference algorithm) [38] have been instrumental in inferring MR proteins representing key functional determinants of tumor-related phenotypes in many cancer types, from glioblastoma [28, 29, 39], neuroblastoma [38], lymphoma [40, 41], and leukemia [42] to prostate [43–45] and breast adenocarcinoma [46–48], among others. We thus reasoned that VIPER could be modified to identify master regulator TKs, most likely to mechanistically regulate the differential phosphorylation pattern observed in a specific tumor sample (**Fig 3A**). A specific additional value of the algorithm is that, as previously shown [28, 38, 43], it could not only identify MR TK proteins, representing individual, pharmacologically accessible dependencies of the tumor, but also TKs representing potential synergistic MR-pair as candidate dependencies for combination therapy.

VIPER can be easily modified to analyze phosphoproteomic signatures (pVIPER). Specifically, rather than assessing the enrichment of a protein’s transcriptional targets (regulon) in differentially expressed genes, pVIPER is designed to measure the enrichment of a TK’s substrates (signalon) in differentially phosphorylated proteins. Since inferred TK-substrate interactions are virtually all positive, this further increases the accuracy of the algorithm by supporting use of a single-tail enrichment analysis as opposed to the three-tail analysis of the original implementation. We first performed pVIPER analysis at the individual phosphopeptide level, rather than by averaging phosphopeptide state on a whole protein level. We then combined the result of the analysis across all phosphopeptides mapping to the same protein. Consistent with VIPER’s experimentally validated ability to identify synergistic master regulators proteins by transcriptional network analysis, pVIPER inferred several candidate synergistic TK interactions based on the statistical significance of the signature-enrichment of substrates shared by both TKs compared to that of substrates uniquely mapped to either one or the other TK (see Method section). Systematic VIPER analysis of phosphoproteomic profiles from 46 LUAD cell lines generated between 2 and 13 master regulator TKs or synergistic TK-pairs, as candidate pharmacologically actionable dependencies, for each cell line, thus generating a plausible number of hypothesis for each cell line (**Fig 3B** and **Fig 3C**).

### pVIPER identifies LUAD-specific dependencies

pVIPER analysis inferred several TK proteins as highly conserved individual dependencies across multiple cell lines, including the Ephrin type-A receptor 2 (*EPHA2*), epidermal growth factor receptor (*EGFR*), c-Met proto-oncogene (*MET*), and HER2 receptor tyrosine kinase 2 (*ERBB2*), suggesting a critical role of these proteins in the maintenance of LUAD cell line state. This is also in agreement with the functional role of these genes and the use of inhibitors of these kinases across a large panel of patients in multiple cancer types [49–53].

In contrast to these established LUAD cell line dependencies, we also identified several TKs as dependencies of specific cell lines. This can either be the result of associated genetic or epigenetic alterations in these cell lines or the result of field effects, where multiple genetic alterations or alterations in upstream pathway contribute to the cell line dependency on a specific TK activity. For instance, we identified ALK (Anaplastic Lymphoma Receptor Tyrosine Kinase) to be addiction point only in H2228 cell line. ALK is a conserved trans-membrane receptor tyrosine kinase (RTK) protein in the insulin-receptor super family. Chromosomal alterations involving *ALK* translocations and fusion events have been identified in several cancer types including LUAD [54, 55], diffuse large B-cell lymphomas [56], neuroblastoma [57], and inflammatory myofibroblastic tumors [58], among others. Additionally, ALK fusion events with other genes, including EML4 (Echinoderm Microtubule-associated protein Like 4) in LUAD lead to aberrant protein activity eliciting “oncogene addiction” [55]. Presence of EML4-ALK fusion transcripts, in ~3–7% of LUAD patients [59–61], is a strong predictor of response to ALK inhibitors, such as Crizotinib, among others [62, 63]. Interestingly, among all available LUAD cell lines for which a phosphoproteomic profile was available, H2228 was the only one with an established EML4-ALK fusion event and with established sensitivity to ALK inhibitor [64, 65]. This further reflects the specificity of our analysis as this was the only cell line predicted to depend on ALK activity. Interestingly, we identified 4 additional H2228 dependencies, namely EGFR, Epha2, c-MET, and PTK2. H2228 sensitivity to EGFR inhibitors, in combination with ALK inhibitors, was already established [65].

### EGFR and c-MET are predicted dependencies in multiple LUAD cell lines

As discussed, pVIPER analysis revealed several TK-pairs as candidate synergistic dependencies across several cell lines, such as Epha2/c-MET, EGFR/PTK2, EGFR/Epha2, Epha2/c-MET, and EGFR/c-MET. Previous study shows that Epha2 blockade could overcome acquired resistance to EGFR kinase inhibitors in Lung Cancer [66]. Among other predicted pairs, the EGFR/c-MET pair emerged as the most conserved synergistic TK-pair across the available cell lines. In addition, EGFR and c-MET were also identified as candidate TK MRs in several of these cell lines, suggesting either a complementary or synergistic role for these proteins and a potential therapeutic opportunity for combination therapy in LUAD [67, 68].

**Fig 4:**
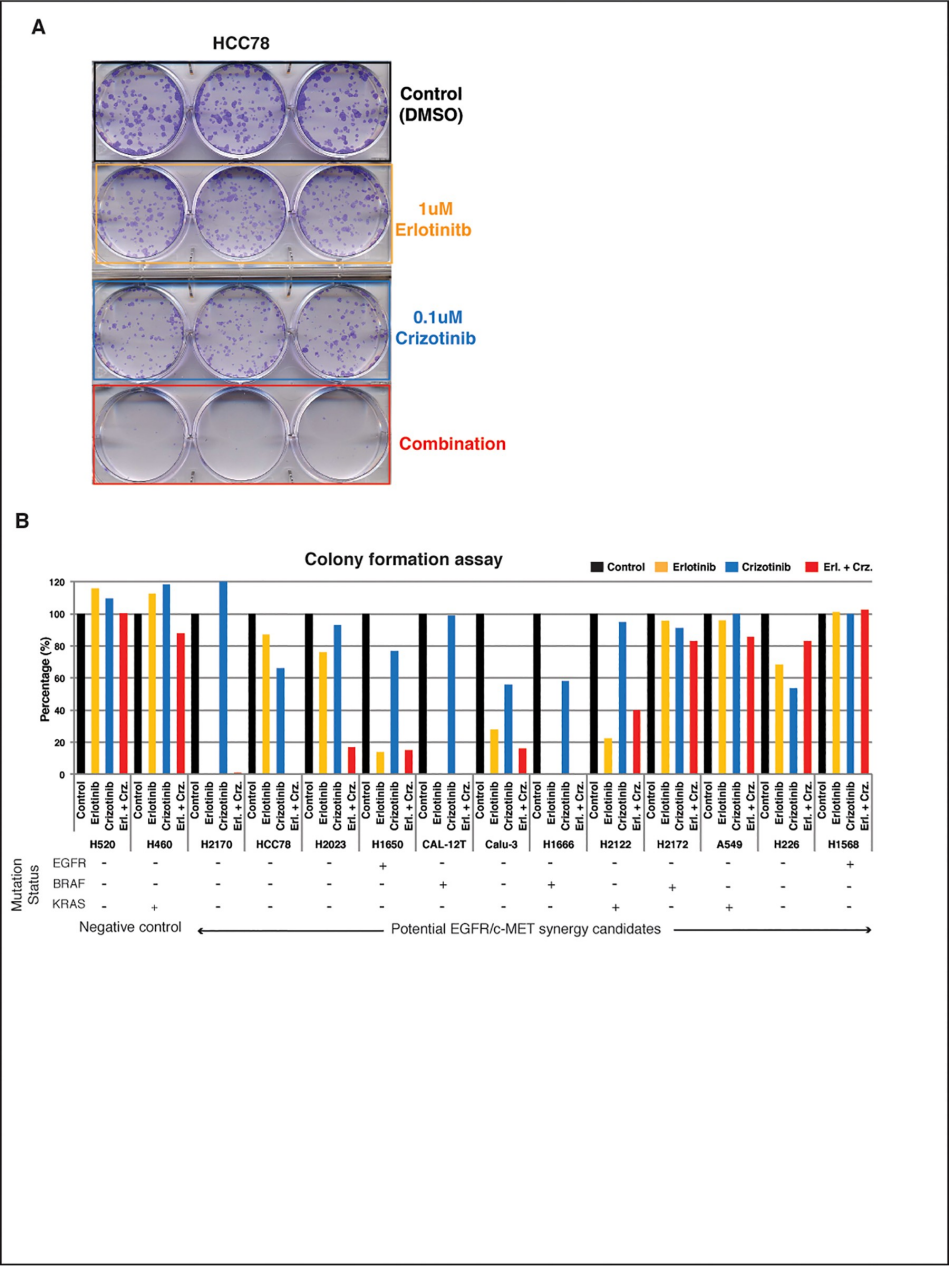
Experimental validation of EGFR and c-MET combination by colony formation assay. **(A)** Colony formation assay schema shows the image of long-term EGFR and c-MET double inhibition effects in HCC78 cell line with different treatments. **(B)** shows long-term colony formation data for 14 cell lines with different EGFR, BRAF and KRAS genomic mutation status.

**Fig 5:**
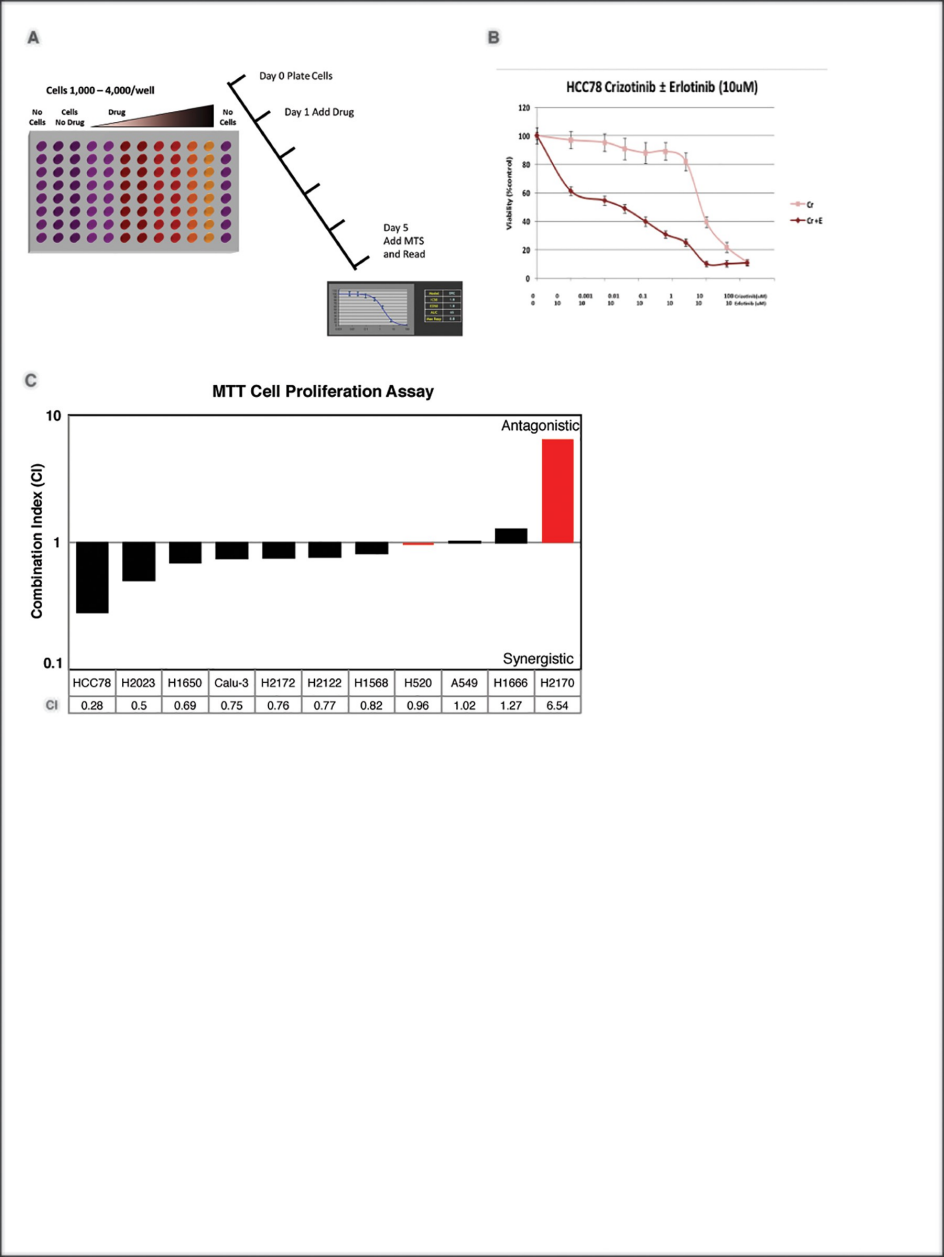
MTT Assay validation of EGFR and c-MET combination. **(A)**. MTT assay experimental schema. **(B)** MTT assay of HCC78 cell line shows synergistic effects of Crizotinib and Erlotinib treatment. **(C)** shows short-term effects of EGFR and c-MET inhibitors’ combination index in 11 cell lines include 2 control cell lines (red).

To validate pVIPER-predicted, cell line specific EGFR/c-MET synthetic lethality, we selected a panel of 14 cell lines, 11 of which were predicted to be synergistically dependent on EGFR/c-MET (H226, H2122, H1666, H2172, Cal-12T, H2023, H1568, Calu-3, H1650, HCC78, and A549), as well as 3 negative controls with no predicted synergistic or individual dependencies on the two TKs (H2170, H460, and H520). To measure sensitivity to these agents, we used two different and complementary assays, including: (a) colony formation assay to assess long term sensitivity (**Fig 4A, S3 Table and Methods**) and (b) 3-[4,5-dimethylthiazol-2-yl]-2,5 diphenyl tetrazolium bromide (MTT) assay for short term sensitivity analysis (**Fig 5A, S4 Table and Methods**). For colony formation assays, cells were treated with either an EGFR inhibitor (Erlotinib, 1uM) or a c-MET/ALK inhibitor (Crizotinib, 0.1uM), either individually or in combination (see Methods). To evaluate synergistic dependency on EGFR/c-MET we used Excess Over Bliss [69], which measures the difference between the observed effect on colony formation and the effect expected from a purely additive model. For MTT assay, first, cells were treated with EGFR inhibitor (Erlotinib) or MET inhibitor (Crizotinib) individually at various concentrations to identify *IC*_*50*_ (concentration resulting in 50% cell death). Next, cells were treated with 1 uM of Erlotinib and varying concentrations of Crizotinib to identify combinations resulting in *IC*_*50*_ and used the combination index (CI) statistic [70] to measure interaction between the two drugs.

Across all 11 cell lines tested in colony formation assay, 8 showed significant sensitivity (Based on half maximal inhibitory concentration, *IC*_*50*_, which represents the concentration of a drug that is required for 50% inhibition) to either individual inhibitors (H226_E,C_, H2122_E_, H1666_E,C_, Cal-12T_E_, Calu-3_E,C_, H1650_E_) or synergistic sensitivity to the combination (HCC78_E+C_ and H2023 _E+C_) (**Fig 4A)**. The sensitivity of a drug was is defined using *IC*_*50*_ according to [71]. Surprisingly, all of these cell lines were EGFR^WT^, ALK^WT^, and c-MET^WT^, except for H1650, which was EGFR^Mut^. Thus, based on standard of care criteria, 7 out of 8 cell lines would not have been considered as sensitive to either EGFR or ALK/c-MET inhibitors. Several cell lines presented striking sensitivity to either one (H2122_E_, Cal-12T_E_, H1650_E_) or both inhibitors (H226_E,C_, H1666_E,C_, Calu-3_E,C_) in isolation, thus making the assessment of synergistic drug sensitivity difficult. In addition, three EGFR^WT^ cell lines harboring BRAF (Cal-12T and H1666) or KRAS (H2122) mutations were also highly sensitive to Erlotinib as a single agent, as predicted by pVIPER, despite the fact that KRAS pathway mutations are mutually exclusive with EGFR mutations and predictive of Erlotinib resistance (**Fig 4B**). Finally, none of these cell lines was predicted to be sensitive to ALK inhibitors, suggesting that Crizotinib sensitivity is mediated by c-MET specific dependencies. Of the negative controls, only one (H2170) showed high sensitivity to Erlotinib. Taken together, 8/11 cell lines (73%) predicted as sensitive to the inhibitors were validated long term colony formation assays, while only 1/3 negative controls showed sensitivity to these agents (33%).

To evaluate the short-term interaction between EGFR and c-MET, we performed MTT assay across 11 cell lines (HCC78, H2023, H1650, Calu-3, H2172, H2122, H1568, A549, H1666, H520 and H2170) including 2 negative control cell lines (H520 and H2170). Similar to colony formation assay, we found synergistic sensitivity to EGFR and c-MET inhibitors in 6/9 cell lines (67%), with 5 cell lines showing strong synergy (CI ≤ 0.8) (**Fig 5B and 5C**) and 1 borderline synergy (CI = 0.82), showing the consistency between two assays. However, for two cell lines, H1666 and H2170 (a negative control), results were inconsistent between long term colony formation and MTT assays. For both H1666 and H2170 cell lines, colony formation and MTT assay to showed sensitivity to Erlotinib alone, where colony formation assay has complete abrogation of colonies at 1 μM of Erlotinib, and later had *IC*_*50*_ = 1.25 μM and 3.7 μM for H1666 and H2170 respectively. However, in combination therapy, MTT assay showed antagonism (CI >1), despite the fact that colony formation assay still showed complete abrogation which could be either the difference in the nature of the two assays or associated to the accumulation of new mutations in these cell lines. However, this is just hypotheses and needs to be verified by further experiments such as sequencing of these cell lines pre-and post-treatment.

### Phosphosite-specific phosphorylation predicts EGFR/c-MET inhibitor synergy

In previous section, we assessed the pVIPER predictions after consolidating the result at protein level. Following the results from MTT and colony formation assays, we reanalyzed the pVIPER predictions at the phosphopeptide level. Interestingly, this revealed that whenever synergistic EGFR/c-MET dependencies were predicted from phosphosite EGFR_1197_ and phosphosites other than c-MET_1003_ (H1666, Cal-12T, H1650), cell lines responded to Erlotinib in isolation, while when predictions were based on phosphosites EGFR_1197_ and c-MET_1003_ (HCC78, H2023, and Calu-3), cells exhibited bona fide synergistic sensitivity to the two inhibitors, with the only possible exception of Calu-3, which showed synergistic sensitivity in MTT assays and additive sensitivity to both inhibitors in colony formation assays. Conversely, when predictions were not based on either phosphotyrosine, cells exhibited no sensitivity to the individual inhibitors or the combination (H2172, H226, A549, H460, H520, H1568). Thus, predictions based on these two phosphosites produced no false positives (6 out of 6 predicted and validated as non-sensitive) and only 2 false negatives (H2170 and H2122), resulting in an error rate of only 2 out of 14 cell lines (14%, p = 0.0093 using fisher exact test).

**Fig 6:**
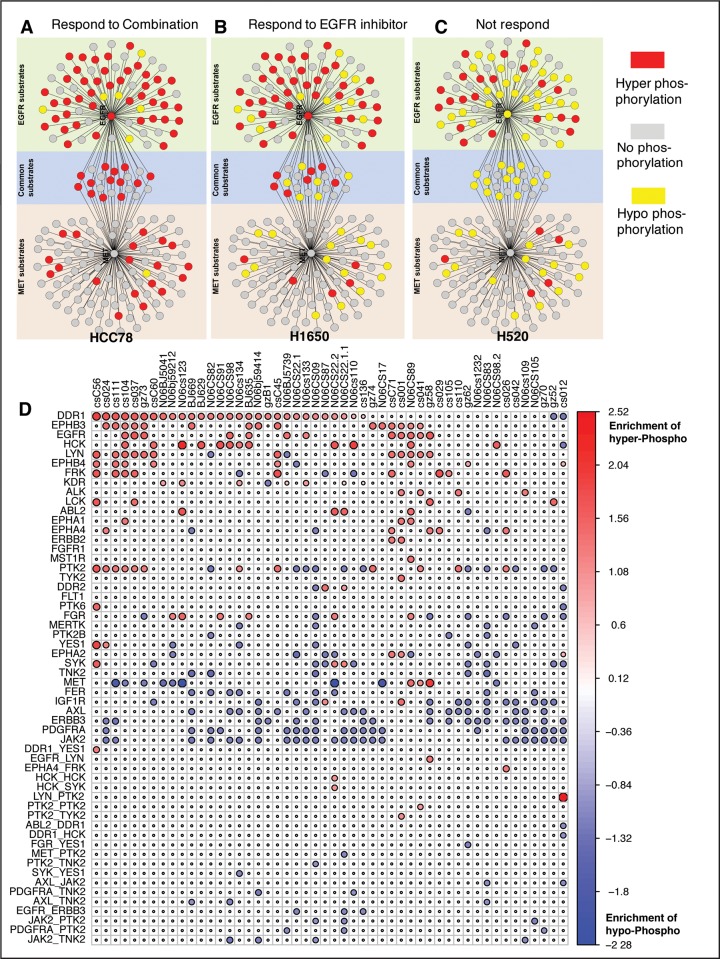
Master regulating peptides in primary lung cancer samples. EGFR and c-MET co-regulate in three scenarios **(A)** when their common substrates are hyperphosphorylated, the patient responds to combination treatment well; **(B)** when most EGFR substrates are hyper-phosphorylated, the patient responds to EGFR inhibitor; **(C)** when substrates of both EGFR and c-MET are mostly hypophosphorylated, the patient does not respond. **(D)** shows the Master Regulator and Master Regulator Pairs regulating hyper/hypo-phosphorylation of their network substrates in each primary samples. In panel (A-C), Red node represent hyper-phosphorylated, yellow node represent hypo-phosphorylated and gray represents un-differentiated phosphorylated proteins compared to average normal samples.

This finding is in agreement with the previously reported finding that the level of tyrosine-1197 phosphorylation on EGFR is predictive of sensitivity [72]. Intriguingly, when sensitivity was predicted using phosphosites other than EGFR_1197_ and c-MET_1003_, cell lines did not respond to the inhibitors, either individually or in combination. For these two peptides, we found their common substrates to be hyper phosphorylated in the sensitive cell lines **(Fig 6A**) compared to the specific substrates of each of them, whereas cell line responding only to EGFR inhibitors showed more hyper phosphorylation of EGFR only substrates (**Fig 6B**). Cell lines resistant to both EGFR and c-MET inhibitors either showed no change in the phosphorylation status or hypo-phosphorylation compared to the normal samples (**Fig 6C**). Hence, either the common substrates of EGFR and c-MET, or the phosphorylation status of EGFR_1197_ and c-MET_1003_ could potentially be used as biomarkers for predicting therapy with the dual inhibitors. However, this conclusion is based on a very limited number of observations and lacks the statistical power. This finding needs a re-evaluation/validation using larger cohort of samples to establish an appropriate biomarker for combination therapy.

### Systematic inference of patient-specific dependencies

Similar to cell lines, when applied on patient data [34], pVIPER identified the tyrosine phosphorylation level of EGFR to be one of the most predictive kinases (**Fig 6**). We inferred EGFR dependency in 12 patients. Of these, 5 harbored EGFR mutations, while the remaining 7 patients had not been tested for this mutation, showing a high consistency between our predictions and the genetic predisposition for sensitivity to EGFR inhibitors. In the entire cohort, there were only 3 patients with EGFR mutations that were not identified as EGFR dependent by pVIPER, resulting in an overall sensitivity of 62.5% (5/8). However, it is well known from literature that >50% of patients harboring EGFR mutations do not respond to Erlotinib [73] suggesting that these may not be false negatives but rather patients with low activation of downstream EGFR pathways, despite their EGFR^Mut^ state. Similarly, our analysis identified candidate ALK dependencies in 4 patients. Of these one had an established TFG-ALK fusion, whereas the others had not been tested for ALK fusion events.

Across all patient samples, we observed Discoidin Domain Receptor-1 (DDR1) to be the most frequent addiction point, which was not predicted for any of the 46 cell lines. One reason for the difference is that DDR1 is collagen dependent and there may be differences in the 3D structure of the tumor and the cell lines growing on the plate. An independent study [74] in a cohort of 83 lung cancer specimens found that silencing of DDR1 in these samples leads to the hampering of cell survival, reduced invasiveness in collagen matrices, increased apoptosis in basal condition and decreased metastatic activity in model of tumor metastasis to bone, signifying it as a potential novel therapeutic target.

## Discussion

In this paper, we developed pARACNe to infer Tyrosine Kinase (TK) signaling network using published global phosphoproteomic data from lung cancer. The network prediction was validated using SILAC experiments, with high accuracy. Interrogation of the predicted TK-substrate network generated biologically meaningful hypotheses, followed by experimental validations illustrating the effectiveness of predicted kinase inhibitor combination, EGFR and c-MET combination inhibitors, in treating lung cancer cell lines. Furthermore, Master Regulator Analysis using patient proteomics data provides implications for using targeted agent combinations to treat patients based on their proteomic profile data.

The new method, *pARACNe*, addresses critical issues that prevented the direct application of the original ARACNe algorithm on phosphoproteomic profile data. Briefly, the new algorithm addresses critical computational challenges presented by LC-MS/MS and spectral counting data, while incorporating enzymatic signaling characteristics into the algorithm design. In particular, pARACNe is designed to handle three critical issues resulting from the use of LC-MS/MS assays, including the highly sparse nature of phosphopeptide abundance data, the large amount of noise and missing data, and the degenerate peptides-to-protein mapping. The algorithm could be applied to proteomics measurements after preprocessing of the original data. For example, where there are datasets from different programs, they could be first transformed into rank based on total peptides identified. When mapping across datasets, one can fill in missing data using 0 –this type of data could be properly handled by the Iterative Quantile Discretization process in pARACNe. Future improvement of pARACNe includes design of a new schema to directly compute Mutual Information from continuous MS/MS data with many missing value produced by multiplexing platforms.

Notably, pARACNe is significant and powerful as of its global scale and context-specificity in discovering global signaling cascading relationships, which were missing by previous methods. For example, methods proposed by Linding et al. [17] combine motif-based phospho-site predictions with information of physical association, co-occurrence, and co-expression to identify substrates with high specificity and accuracy, but with low coverage and lack of contextual specificity. Bender et al [18] used reverse phase protein assay data after various stimulations to cells and inferred signaling network using hidden Markov models and genetic algorithms. Even though the resulting networks are context specific, they lack genomic-scale coverage. There have been methods which used existing large-scale protein networks and prune them using transcriptomic information to identify signaling pathways [75–77]. In addition, attempts have been made to reconstruct signaling network using gene expression data [78, 79]. However, as signaling complexity lies mostly in upper level of cellular processes, inferring the cascades from downstream gene expression fails to capture all the dynamics. Also, PrePPI proposed by Zhang et al. [80] used protein structure-based methods to infer global protein-protein interaction, but this approach fails to address phosphorylation context specificity. Innovative uses of multiplex and microarray-based approaches, where multiple antibodies can be used to probe an ensemble of phosphoproteins, are finally becoming sufficiently mature to allow characterization of small pathways. Yet, these methods are still far from providing an unbiased, global view of tyrosine signal-transduction processes and continue to be completely dependent on antibody specificity and availability. Similarly, assays that are generally used to monitor phosphorylation pathways, such as Stable Isotope Labeling with Amino acids in Cell culture (SILAC), provide a simple and straightforward approach to detect differential protein abundance. Coupled with phosphorylation enriched assays, it can provide high quality quantification for post-translation phosphorylation changes in cell lines. However, these methods are 1) laborious and costly; 2) can only be performed to dissect the substrates of a single enzyme at a time and 3) do not differentiate between direct and indirect targets. Recently developed TMT/iTRAQ experiments uses multiplexing strategy to investigate multiple treatments or Kinase knock-out/knock-down experiments [81, 82], thus making the process high-throughput at reduced the cost, however still cannot provide evidence for direct targets.

To be noted, as the LC-MS/MS experiments used here was generated based on Tyrosine-kinase enrichment, which is only about ~2% of whole phosphoproteome. pARACNe is shown only on TK-substrates network. The current methodology could be extended to whole phosphoproteomic data based signaling network reconstruction where the data is available. In addition to label free based LC-MS/MS proteomics data used in this work, label-based approaches, such as ITRAQ or TMT, could generate higher throughput whole proteomic profiles which might require future redesign of ARACNe to incorporate both kinases and phosphatases in regulating their downstream substrates. It is reasonable to expect that a version of ARACNe developed specifically to dissect signaling networks should work at least as well as its transcriptional counterpart. Since the relationship between the mRNA abundance of a gene encoding a transcription factor (TF) and the activity of the corresponding protein is much looser than that between the abundance of a phospho-isoform of a kinase and its enzymatic activity.

Even though research has attempted to identify addiction points based on gene expression data [83], predictions based on phosphoproteomic data appear superior in a way that they can directly reflect contextual specific signaling activity and are able to be directly targeted by kinase inhibitors. It is important to note that clinically, only patients with base-pair deletion at exon 19 (del746_A750) or a point mutation at exon 21 mutation (L858R) in EGFR shows sensitivity to EGFR inhibitor such as Cetuximab or Erlotinib [84]. In the future, whole phosphoproteomic data and tools could be used in combination with transcriptome-based approaches to reveal protein/gene biomarkers for patient stratification and additional target for personalized therapy.

## Methods and data

### Phosphoproteomic data

The previously published phosphoproteomic data used to reconstruct signaling network was download from [54]. This dataset, representing the abundance of phospho-tyrosine containing peptides, was obtained by tandem mass spectrometry analysis of 46 non-small cell lung cancer (NSCLC) cell lines, 151 NSCLC tumors, and 48 normal lung tissue samples. Immunohistochemistry and a phospho-tyrosine specific antibody were used to screen 96 paraffin-embedded, formalin fixed tissue samples from NSCLC patients as described by Rikova et al.[54]. About 30% of tumors showed high-levels of phospho-tyrosine expression. Immunoblotting of 46 NSCLC cell lines with a phospho-tyrosine specific antibody also showed heterogeneous reactivity especially in the molecular weight range characteristic of receptor tyrosine kinases.

Since phospho-tyrosine represents less than 1% of the cellular phosphoproteome, as determined by tandem mass spectrometry (MS/MS), and is difficult to analyze by conventional methods, immuno-affinity purification was performed with a phospho-tyrosine antibody to enrich for phospho-tyrosine containing peptides prior to tandem mass spectrometry. All tumors were identified as NSCLC based on standard pathology. Only those tumors with greater than 50% of cancer cells were considered for further analysis. NSCLC cell lines were grown overnight in low serum to reduce background phosphorylation from culture conditions.

Tandem MS profiling identified 3920 tyrosine phosphorylation sites on approximately 2600 different proteins. 85% of these sites appeared to be novel when compared against PhosphoSite (http://www.phosphosite.org), a comprehensive resource of known phosphorylation sites.

### pARACNe algorithm

ARACNe is originally designed for gene expression data, where expression of genes is usually continuous and non-sparse. Quantitative data obtained from label-free LC-MS/MS by data-dependent acquisition via spectral counting is discrete and very sparse, with many phosphopeptides counts not observed for multiple peptides in each sample causing the current version of ARACNe to be not suitable for this data, which thus required major modifications to handle discrete data. To handle discrete abundances, we modified the mutual information computation approach from a kernel density estimation based method to a Naïve based estimation of mutual information, which is a histogram based technique[85]. Briefly, consider a collection of *N* simultaneous measurements of two genes X and Y. Data is partitioned into M discrete bins *a*_*i*_, and *k*_*i*_ denotes the number of measurements that lie within the bin *a*_*i*_. The probabilities *p*(*a*_*i*_) are then approximated by the corresponding relative frequencies of occurrence $p(a_{i})\to \frac{k_{i}}{N}$ and the mutual information *I*(*X*,*Y*) between datasets X and Y is expressed as

$$I(X,Y)=\log N+\frac{1}{N}\sum_{ij}k_{ij}\log\frac{k_{ij}}{k_{i}k_{j}}$$

Here *k*_*ij*_ denotes the number of measurements where X lies in *a*_*i*_ and Y in *a*_*j*_ and *N* total number of samples.

Accuracy of mutual information is dependent on correct numbers of bins, M. To find the optimal number of bins we applied ARACNe on the whole dataset by varying M from 1 to 20 and testing the connections in predicted sub-network against the set of known connections (gold standard) from databases (phosphoDB) [35].

In case of continuous data, partitioning can be achieved by dividing the range of data into M equally spaced distance bins. Our data being discrete, equally spaced partitioning was not possible. So, to overcome this problem, we used an iterative approach of partitioning **(Fig 1C)**. The basic idea is to divide the number of N data points into M with each bin containing equal number of data point. If the data point(s) with the same value falls into consecutive bin(s), we put those data point(s) into current bin and repartition the remaining points into remaining number of bins. We keep iterating this till we finish either the bins or there are no more data points to bin. For example, in **Fig 1C** step (3), we initially partition N points into 4 bins. The data points with 0 value does not fit into first bin and falls into subsequent bins, so we assign all data points with 0 value into first bin and repartition the remaining points into 3 bins. We keep on doing this process till we achieve 4 bins.

To evaluate initial performance and decide number of bins, we computed the network among all tyrosine kinases and substrates, parsed the sub-network between 49 tyrosine kinases and 114 substrates which were present in PhosphoSite database and compared the results with the connections present in database. From our analysis, we found that M = 10 to be an optimal number **(S1 Fig)** which gave us precision of 14% and sensitivity of 24%. This precision is an underestimate of real precision as in the gold standard many interactions are not present.

### Master regulator analysis

To discover the master regulator in various cell lines, we interrogated the network obtained from pARACNe using a novel algorithm, VIPER (Virtual Proteomics by Enriched Regulon analysis) [23], designed to infer kinases that are key players in a particular cell line. Protein activity is a good indicator of key kinases in a particular phenotype but often phosphorylated state of a protein is not sufficient to determine its activity both due to measurement noise in phosphorylated state as well as LC-MS/MS technique noise. To overcome this problem, VIPER infers kinase activity from the global kinase substrate relationship and its biological relevance by overlapping this information in a particular phenotype-specific program.

VIPER requires a network model and signature of the phenotype transition (i.e., all genes ranked by their differential phosphorylation in two phenotypes). Here, the signature, *S*_*kin*_, was obtained by t-test analysis by comparing each cell line against all normal samples. First, we associate each kinase with positive and negative activity targets, by computing the correlation between each kinase and its predicted substrates and selecting only those substrates which had a significant correlation (p-value ≤ 0.05, Bonferroni corrected). Second, for each kinase we computed an *activity* by measuring the enrichment of the *S*_*kin*_ signature in predicted substrates list, separately for both positive and negative correlated, (*S*_*kin*_*-enrichment*). Enrichment was computed by Gene Set Enrichment Analysis (GSEA). Since very small percentage of kinases are found to have negative correlation, we did not use those interactions to evaluate enrichment.

### Cell culture

All cell lines were grown in RPMI-1640 with 5% fetal bovine serum and incubated at 37°C in a humidified atmosphere containing 5% CO_2_. Cell lines were fingerprinted using the Perplex 1.2 system (Promega, Madison, WI). Fingerprints were compared to those generated at ATCC and/or our internal database.

### MTT assays

Short term MTT assays (CellTiter 96 AQ_ueous_ One Solution Cell Proliferation Assay, Promega, Madison WI) were performed as previously described in [86]. Specifically, each drug concentration is octuplicated and the mean with standard deviation of all replicates were used to generate a curve to allow calculation of the drug *IC*_*50*_ (Inhibitory Concentration of 50%) value. The assays were repeated at least 3 times and the *IC*_*50*_s are the average of all replicates.

### Colony-formation assays

Long term colony formation assays were performed in triplicate in 6-well plates. Cells were added to media containing drug and incubated for 1–2 weeks such that control wells (no drug) contained colonies of 50–70 cells each. At such time media was removed and all wells stained with a solution containing 0.5% crystal violet and 6% glutaraldehyde for 1 hour. The plates were then rinsed, dried, and colonies were manually counted.

### SILAC experiments

EGFR SILAC experiment was performed in H3255 cell line by treating samples with Gefitinib. c-MET SILAC experiment was performed in c-MET-driven gastric cancer cell line, MKN45, by using c-MET inhibitor Su11274. For both genes, cells were treated with inhibitors for 3 and 24hr. For control, cells were grown in same conditions but were not treated with the drug. For our comparison we combined the peptides, which were differentially obtained between treated and untreated samples, for 3 and 24 hr. More details about the experiment can be obtained from Guo et al [34].

#### Gold standard

In PhosphoSite database, there were 282 connections between 49 tyrosine kinases and 114 substrates.

## Supporting information

S1 Fig Performance of the pARACNe algorithm. To select optimal bin number in pARACNe algorithm, precision and recall curves for various number of bins were computed. Black curve is when no binning of data is done. When using 10 bins, the algorithm achieved the best performance.
(PDF)

S1 Table pARACNe-inferred TK-peptides/substrate-peptides Interaction Network. A three-column table. Column one and two are the two interacting peptides. Column one represents the gene symbol with dollar sign ($) and numbers specify the sites of phosphorylation in the protein. Column two is the substrate, representing a gene symbol followed by underscore and a number of phosphorylation sites. Column three is the mutual information between the two peptides in column one and column two.
(XLSX)

S2 Table pARACNe-inferred TK-Protein/Substrate Interaction Network. A three column table includes kinase, mutual information and substrate. Column two is the maximum mutual information when aggregating the peptide network into protein level network.
(XLSX)

S3 Table Colony formation assay result. The results for colony formation assay. First column is the cell line, second column is the treatment used, and third column is the mean number of colonies (from triplicates) after normalizing to the control group for each cell line.
(XLSX)

S4 Table MTT assay results. The results for MTT assay. First column is the cell line, second column is the IC_50_ for Erlotinib, third Column is the IC_50_ for Crizotinib, fourth column is the IC_50_ of Crizotinib when cell cells were treated with 1 uM of Erlotinib and varying concentrations of Crizotinib and fifth column is the combination index (CI).
(XLSX)
