# Supplementary figures and images for: Elucidating synergistic dependencies in lung adenocarcinoma by proteome-wide signaling-network analysis
Source: PLoS One. 2019 Jan 7;14(1):e0208646. doi: 10.1371/journal.pone.0208646 (PMC6322741; doi:10.1371/journal.pone.0208646)

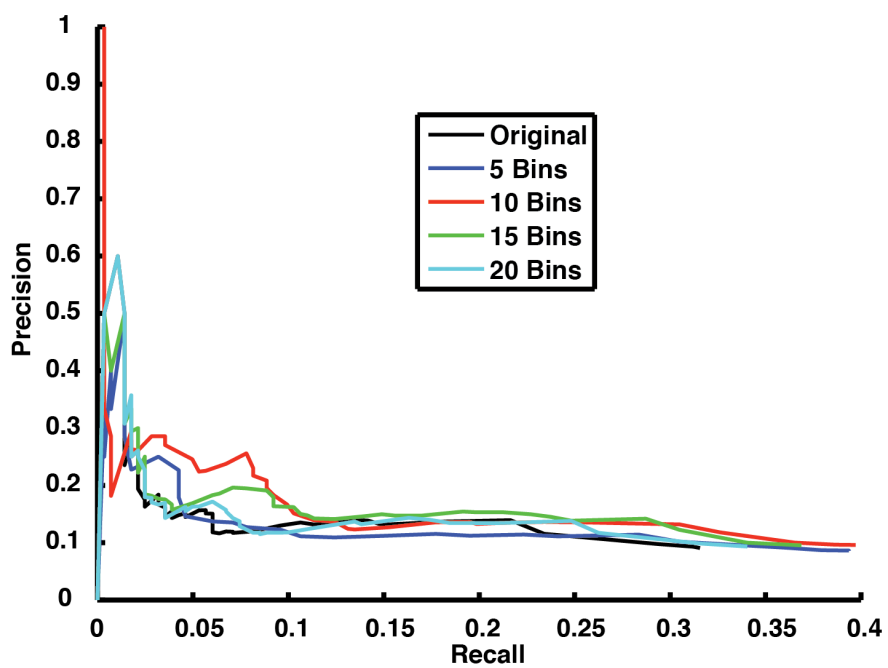

Supplement: S1 Fig — To select optimal bin number in pARACNe algorithm, precision and recall curves for various number of bins were computed. Black curve is when no binning of data is done. When using 10 bins, the algorithm achieved the best performance. (PDF) [file pone.0208646.s001.pdf]
